# West Nile Virus and Other Nationally Notifiable Arboviral Diseases — United States, 2021

**DOI:** 10.15585/mmwr.mm7234a1

**Published:** 2023-08-25

**Authors:** Anna C. Fagre, Shelby Lyons, J. Erin Staples, Nicole Lindsey

**Affiliations:** ^1^Division of Vector-Borne Diseases, National Center for Emerging and Zoonotic Infectious Diseases, CDC; ^2^Epidemic Intelligence Service, CDC.

**Summary**

**What is already known about this topic?**

West Nile virus (WNV), transmitted primarily through mosquitos, is the leading cause of arboviral disease in the continental United States, but other arboviruses cause sporadic cases of neuroinvasive disease.

**What is added by this report?**

Arizona experienced a significant WNV outbreak in 2021, with three counties reporting more than 50% of all reported WNV cases nationwide. Nationally, the rate of WNV neuroinvasive disease (0.61 per 100,000 population) surpassed the median rate during 2010–2020 (0.39) and was the highest since 2012.

**What are the implications for public health practice?**

Health care providers should consider arboviral infections in the differential diagnosis of aseptic meningitis and encephalitis, obtain appropriate specimens for laboratory testing, and promptly report cases to public health authorities.

## Abstract

Arthropod-borne viruses (arboviruses) are transmitted to humans primarily through the bites of infected mosquitoes or ticks, and in the continental United States, West Nile virus (WNV) is the leading cause of domestically acquired arboviral disease. Other arboviruses cause sporadic cases of disease as well as occasional outbreaks. This report summarizes 2021 surveillance data reported to CDC by U.S. jurisdictions for nationally notifiable arboviruses; the report excludes chikungunya, dengue, yellow fever, and Zika virus disease cases, because these infections were acquired primarily through travel during 2021. Forty-nine states and the District of Columbia reported 3,035 cases of domestic arboviral disease, including those caused by West Nile (2,911), La Crosse (40), Jamestown Canyon (32), Powassan (24), St. Louis encephalitis (17), unspecified California serogroup (six), and eastern equine encephalitis (five) viruses. Among the WNV disease cases, 2,008 (69%) were classified as neuroinvasive disease, for a national incidence of 0.61 cases per 100,000 population. Because arboviral diseases continue to cause serious illness, maintaining surveillance programs to monitor their transmission and prevalence is important to the direction and promotion of prevention activities. Health care providers should consider arboviral infections in the differential diagnosis of aseptic meningitis and encephalitis, obtain appropriate specimens for laboratory testing, and promptly report cases to public health authorities. Prevention depends on community and household efforts to reduce vector populations and personal protective measures to prevent mosquito and tick bites, such as use of Environmental Protection Agency–registered insect repellent and wearing protective clothing.

## Introduction

Within the continental United States, West Nile virus (WNV) is the leading cause of domestically acquired disease caused by arthropod-borne viruses (arboviruses) (*1*). Arboviruses are maintained in transmission cycles between arthropods and vertebrate hosts, including humans and other animals (*2*). Humans primarily become infected when they are bitten by an infected tick or mosquito. Whereas most human infections are asymptomatic, symptomatic infections commonly manifest as a systemic febrile illness and less commonly as neuroinvasive disease.

## Methods

Most domestic arboviral diseases are nationally notifiable and reported by state health departments to CDC through the national arboviral surveillance system (ArboNET) using standard surveillance case definitions that include clinical and laboratory criteria.* Confirmed^†^ and probable^§^ cases are included in this analysis. Cases reported as meningitis, encephalitis, acute flaccid paralysis, or other neurologic illnesses are classified as neuroinvasive disease; the remainder are considered nonneuroinvasive disease. Incidence was calculated using neuroinvasive disease cases and the U.S. Census Bureau’s 2021 midyear population estimates.^¶^ All statistical analyses were conducted using SAS software (version 9.4; SAS Institute). This activity was reviewed by CDC and was conducted consistent with applicable federal law and CDC policy.**

## Results

In 2021, a total of 3,035 domestic arboviral disease cases were reported to CDC. Among these, 2,113 (70%) were neuroinvasive. Cases were caused by the following viruses: WNV (2,911 cases; 96%), La Crosse (40; 1%), Jamestown Canyon (32; 1%), Powassan (24; 1%), St. Louis encephalitis (17; 1%), unspecified California serogroup (six; <1% [exact virus unknown]), and eastern equine encephalitis (five; <1%). Except for Hawaii, cases were reported from all states and the District of Columbia. Among the 3,143 U.S. counties, 500 (16%) reported at least one case of arboviral disease.

### West Nile Virus Disease

Cases of WNV disease were reported from 432 counties in 49 states and the District of Columbia. Most (71%) patients had illness onset during July–September (Table 1). The median patient age was 65 years (IQR = 52–74 years), and 1,739 (60%) were male. A total of 2,099 (72%) patients were hospitalized, and 227 (8%) died. The median age of patients who died was 75 years (IQR = 68–82 years).

**TABLE 1:** Number and percentage of reported cases of nonneuroinvasive and neuroinvasive West Nile virus and other arboviral diseases, by virus type and selected patient characteristics — United States, 2021*

Characteristic	Virus type, no. of cases (%)					
	West Nile (n = 2,911)	La Crosse (n = 40)	Jamestown Canyon (n = 32)	Powassan (n = 24)	St. Louis encephalitis (n = 17)	Eastern equine encephalitis (n = 5)
**Age group, yrs**						
<18	38 (1)	35 (88)	1 (3)	3 (13)	0 (—)	0 (—)
18–59	1,055 (36)	3 (8)	16 (50)	5 (21)	2 (12)	2 (40)
≥60	1,817 (62)	2 (5)	15 (47)	16 (67)	15 (88)	3 (60)
Unknown	1 (<1)	0 (—)	0 (—)	0 (—)	0 (—)	0 (—)
**Median age (IQR)**	65 (52–74)	6 (5–11)	59 (41–71)	68 (48–75)	73 (63–76)	61 (57–61)
**Sex**						
Female	1,172 (40)	12 (30)	6 (19)	11 (46)	4 (24)	3 (60)
Male	1,739 (60)	28 (70)	26 (81)	13 (54)	13 (76)	2 (40)
**Period of illness onset**						
Jan–Mar	5 (<1)	1 (3)	0 (—)	0 (—)	0 (—)	0 (—)
Apr–Jun	34 (1)	5 (13)	9 (28)	13 (54)	0 (—)	0 (—)
Jul–Sep	2,067 (71)	32 (80)	18 (56)	5 (21)	6 (35)	3 (60)
Oct–Dec	705 (24)	2 (5)	5 (16)	6 (25)	11 (65)	2 (40)
Unknown	100 (3)	0 (—)	0 (—)	0 (—)	0 (—)	0 (—)
**Clinical syndrome**						
Nonneuroinvasive	903 (31)	1 (3)	11 (34)	1 (4)	6 (35)	0 (—)
Neuroinvasive	2,008 (69)	39 (98)	21 (66)	23 (96)	11 (65)	5 (100)
Encephalitis^†^	1,276 (64)	31 (78)	13 (41)	12 (50)	7 (41)	5 (100)
Meningitis^†^	602 (30)	8 (20)	4 (13)	8 (33)	4 (24)	0 (—)
AFP^†,§^	42 (2)	0 (—)	1 (3)	3 (13)	0 (—)	0 (—)
Unspecified^†^	88 (4)	0 (—)	3 (9)	0 (—)	0 (—)	0 (—)
**Outcome**						
Hospitalization	2,099 (72)	40 (100)	24 (75)	22 (92)	15 (88)	5 (100)
Death	227 (8)	0 (—)	2 (6)	3 (13)	0 (—)	2 (40)

**Abbreviation:** AFP = acute flaccid paralysis.

* Six unspecified California serogroup virus cases were also reported.

^†^ Percentages of case of encephalitis, meningitis, AFP, and unspecified neurologic signs or symptoms are percentages of neuroinvasive cases.

^§^ Among the 42 West Nile virus disease cases with AFP, 12 (29%) also had encephalitis or meningitis.

Among the 2,008 WNV neuroinvasive disease cases, 1,276 (64%) were reported as encephalitis, 602 (30%) as meningitis, 42 (2%) as acute flaccid paralysis, and 88 (4%) as unspecified neurologic illness. Twelve (29%) of the 42 patients with acute flaccid paralysis also had encephalitis or meningitis. Most patients with neuroinvasive disease (1,907; 95%) were hospitalized and 225 (11%) died. Nationally, the incidence of neuroinvasive WNV disease was 0.61 per 100,000 population (Table 2), and the proportion of cases classified as neuroinvasive in 2021 (69%) was higher than the average proportion of cases classified as neuroinvasive during 2010–2020 (63%).^††^

**TABLE 2:** Number and rate* of reported cases of arboviral neuroinvasive disease, by virus type and U.S. Census Bureau division and jurisdiction — United States, 2021

U.S. Census Bureau division/ Jurisdiction	Neuroinvasive disease cases, by virus type, no. (incidence)*					
	West Nile	La Crosse	Jamestown Canyon	Powassan	St. Louis encephalitis	Eastern equine encephalitis
**United States**	**2,008 (0.61)**	**39 (<0.01)**	**21 (<0.01)**	**23 (<0.01)**	**11 (<0.01)**	**5 (<0.01)**
**New England**	**17 (0.11)**	**—^†^**	**6 (0.04)**	**13 (0.09)**	**—**	**—**
Connecticut	5 (0.14)	—	—	3 (0.08)	—	—
Maine	—	—	1 (0.07)	3 (0.22)	—	—
Massachusetts	9 (0.13)	—	—	6 (0.09)	—	—
New Hampshire	1 (0.07)	—	4 (0.29)	—	—	—
Rhode Island	1 (0.09)	—	1 (0.09)	1 (0.09)	—	—
Vermont	1 (0.15)	—	—	—	—	—
**Middle Atlantic**	**83 (0.20)**	**—**	**1 (<0.01)**	**2 (<0.01)**	**—**	**—**
New Jersey	26 (0.28)	—	1 (0.01)	—	—	—
New York	35 (0.18)	—	—	2 (0.01)	—	—
Pennsylvania	22 (0.17)	—	—	—	—	—
**East North Central**	**116 (0.25)**	**22 (0.05)**	**10 (0.02)**	**3 (<0.01)**	**—**	**2 (<0.01)**
Illinois	49 (0.39)	1 (<0.01)	—	—	—	—
Indiana	11 (0.16)	1 (0.01)	1 (0.01)	—	—	—
Michigan	38 (0.38)	1 (<0.01)	5 (0.05)	—	—	1 (<0.01)
Ohio	11 (0.09)	17 (0.14)	—	1 (<0.01)	—	—
Wisconsin	7 (0.12)	2 (0.03)	4 (0.07)	2 (0.03)	—	1 (0.02)
**West North Central**	**151 (0.70)**	**—**	**4 (0.02)**	**5 (0.02)**	**—**	**—**
Iowa	4 (0.13)	—	—	—	—	—
Kansas	10 (0.34)	—	—	—	—	—
Minnesota	27 (0.47)	—	4 (0.07)	5 (0.09)	—	—
Missouri	11 (0.18)	—	—	—	—	—
Nebraska	69 (3.51)	—	—	—	—	—
North Dakota	12 (1.55)	—	—	—	—	—
South Dakota	18 (2.01)	—	—	—	—	—
**South Atlantic**	**38 (0.06)**	**10 (0.02)**	**—**	**—**	**—**	**3 (<0.01)**
Delaware	3 (0.30)	—	—	—	—	—
District of Columbia	4 (0.60)	—	—	—	—	—
Florida	5 (0.02)	—	—	—	—	—
Georgia	3 (0.03)	—	—	—	—	2 (0.02)
Maryland	7 (0.11)	—	—	—	—	—
North Carolina	8 (0.08)	9 (0.09)	—	—	—	1 (<0.01)
South Carolina	6 (0.12)	—	—	—	—	—
Virginia	2 (0.02)	—	—	—	—	—
West Virginia	—	1 (0.06)	—	—	—	—
**East South Central**	**21 (0.11)**	**7 (0.04)**	**—**	**—**	**—**	**—**
Alabama	9 (0.18)	—	—	—	—	—
Kentucky	4 (0.09)	—	—	—	—	—
Mississippi	5 (0.17)	—	—	—	—	—
Tennessee	3 (0.04)	7 (0.10)	—	—	—	—
**West South Central**	**172 (0.42)**	**—**	**—**	**—**	**1 (<0.01)**	**—**
Arkansas	6 (0.20)	—	—	—	—	—
Louisiana	17 (0.37)	—	—	—	1 (0.02	—
Oklahoma	19 (0.48)	—	—	—	—	—
Texas	130 (0.44)	—	—	—	—	—
**Mountain**	**1,308 (5.18)**	**—**	**—**	**—**	**6 (0.02)**	**—**
Arizona	1,140 (15.67)	—	—	—	6 (0.08)	—
Colorado	101 (1.74)	—	—	—	—	—
Idaho	12 (0.63)	—	—	—	—	—
Montana	2 (0.18)	—	—	—	—	—
Nevada	1 (0.03)	—	—	—	—	—
New Mexico	31 (1.47)	—	—	—	—	—
Utah	21 (0.63)	—	—	—	—	—
Wyoming	—	—	—	—	—	—
**Pacific**	**102 (0.19)**	**—**	**—**	**—**	**4 (<0.01)**	**—**
Alaska	1 (0.14)	—	—	—	—	—
California	96 (0.24)	—	—	—	4 (0.01)	—
Hawaii	—	—	—	—	—	—
Oregon	1 (0.02)	—	—	—	—	—
Washington	4 (0.05)	—	—	—	—	—

* Cases per 100,000 population, based on July 1, 2021, U.S. Census Bureau population estimates.

^†^ Dashes indicate no cases were reported.

Arizona reported the largest number of neuroinvasive cases (1,140) and the highest incidence of WNV neuroinvasive disease (15.7 per 100,000 population). Three counties in Arizona (Maricopa, Pima, and Pinal) accounted for >50% of WNV neuroinvasive disease cases nationwide. Jurisdictions with the next highest numbers of WNV disease cases were Texas (130), Colorado (101), California (96), and Nebraska (69). Incidence of neuroinvasive WNV disease was highest in several states within the Mountain and West North Central U.S. Census Bureau divisions^§§^ (Figure). Incidence increased with age, from 0.02 per 100,000 population among children aged <10 years to 2.4 among adults aged ≥70 years. Incidence was 60% higher overall among males (0.8) than among females (0.5).

**FIGURE:**
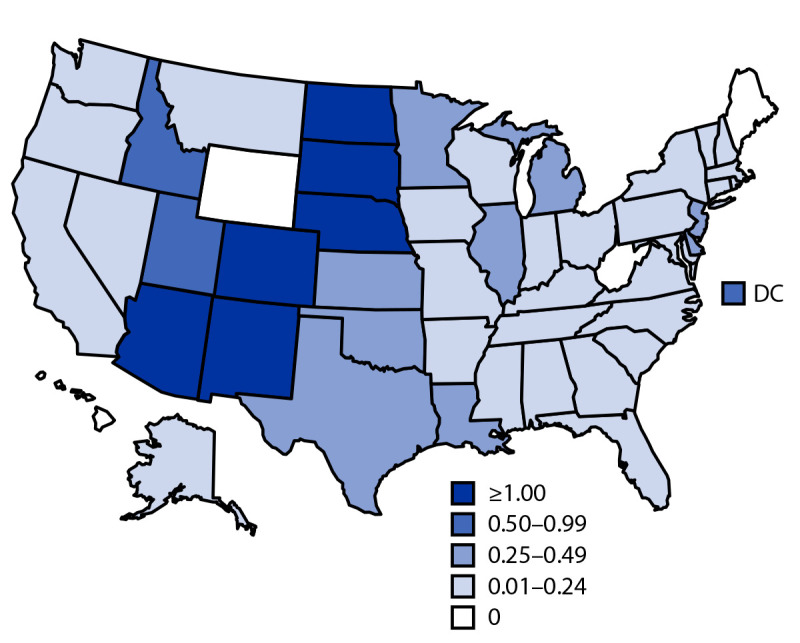
Incidence* of reported cases of West Nile virus neuroinvasive disease — United States, 2021 **Abbreviation:** DC = District of Columbia. * Cases per 100,000 population based on July 1, 2021, U.S. Census Bureau population estimates.

### La Crosse Virus Disease

Forty La Crosse virus disease cases were reported from eight jurisdictions. Jurisdictions with the highest neuroinvasive disease incidence included Ohio (0.14 per 100,000 population), Tennessee (0.10), and North Carolina (0.09) (Table 2). The median patient age was 6 years (IQR = 5–11 years), and 35 (88%) cases occurred among persons aged <18 years (Table 1). Most patients (80%) had illness onset during July–September. All 40 patients were hospitalized, and 98% had neuroinvasive disease; no deaths were reported.

### Jamestown Canyon Virus Disease

Thirty-two Jamestown Canyon virus disease cases were reported from eight jurisdictions. Jurisdictions with the highest neuroinvasive disease incidence included New Hampshire (0.29 per 100,000 population) and Rhode Island (0.09) (Table 2). In 2021, Jamestown Canyon virus disease was reported from Indiana for the first time. The median patient age was 59 years (IQR = 41–71), and 26 (81%) patients were male (Table 1). Illness onset occurred during April–November, with 18 (56%) patients reporting onset during July–September. Twenty-one (66%) cases were neuroinvasive, and 24 (75%) patients were hospitalized. Two (6%) patients died, both of whom were aged >60 years.

### Powassan Virus Disease

Twenty-four Powassan virus disease cases were reported from eight jurisdictions, with highest neuroinvasive disease incidence in the New England (0.09 per 100,000 population) and West North Central (0.02) (Table 2) U.S. Census Bureau divisions. Powassan virus disease was reported for the first time from Ohio. The median patient age was 68 years (IQR = 48–75) and 13 (54%) were male (Table 1). Illness onset dates occurred during April–November, with 13 (54%) reporting onset during April–June. Twenty-three (96%) cases were neuroinvasive, and 22 (92%) patients were hospitalized. Three (13%) patients died, including two who were aged >60 years.

### St. Louis Encephalitis Virus Disease

Seventeen cases of St. Louis encephalitis virus disease were reported from three states: Arizona (12), California (four), and Louisiana (one) (Table 1). Eleven (65%) cases were neuroinvasive (Table 2). Among all cases, the median patient age was 73 years (IQR = 63–76 years), and 13 (76%) were male (Table 1). Illness onset dates occurred during August–November, with 11 (65%) patients reporting onset during October–December. Fifteen (88%) patients were hospitalized, and none died.

### Eastern Equine Encephalitis Virus Disease

Five cases of eastern equine encephalitis virus disease were reported from four states: Georgia (two), Michigan (one), North Carolina (one), and Wisconsin (one) (Table 2). The median patient age was 61 years (IQR = 57–61 years), and two (40%) were male. Illness onset dates occurred during July–October. All cases were neuroinvasive and resulted in hospitalization. Two (40%) patients died; both were aged <60 years.

## Discussion

As in previous years, WNV was the most common cause of neuroinvasive arboviral disease in the United States in 2021, accounting for 95% of reported neuroinvasive arboviral disease cases. The incidence of WNV neuroinvasive disease (0.61 per 100,000 population) surpassed the median during 2010–2020 (0.39) and was the highest since 2012 (0.92) (*3*,*4*). This increase was largely driven by a significant outbreak in Arizona (1,140 cases; 57% of all U.S. cases)^¶¶^ (*5*), concentrated in Maricopa, Pinal, and Pima counties. Compared with previous years, the outbreak occurred later in the year, increasing the proportion of patients with illness onset during October–December. Reasons for the outbreak likely included late-season rain, recent population growth and housing development, and low levels of WNV circulation during the preceding year (*1*,*5*).

La Crosse virus continued to be the most common cause of neuroinvasive arboviral disease in children. In 2021, Jamestown Canyon virus was reported for the first time in Indiana, and Powassan virus was reported for the first time in Ohio (*6*,*7*). Detection of these viruses in new jurisdictions is likely caused by an increase in awareness and testing but could also reflect geographic expansion of these pathogens.

Although case numbers vary by year, virus, and geographic area, arboviruses continue to cause substantial morbidity in the United States. Weather, zoonotic host and vector abundance, and human behavior are all factors that can influence when and where outbreaks occur. This complexity makes it difficult to predict future locations and timing of cases and underscores the importance of surveillance to identify outbreaks quickly to direct public health prevention efforts.

### Limitations

The findings in this report are subject to at least two limitations. First, because ArboNET does not require information about clinical signs and symptoms or laboratory findings, cases might be misclassified. Second, ArboNET is a passive surveillance system that only collects cases that are diagnosed and reported, resulting in underestimation of the actual incidence of disease. The COVID-19 pandemic likely exacerbated this trend (*8*); the percentage of cases classified as neuroinvasive disease during 2021 (69%) was higher than that reported during 2010–2020 (63%), indicating an underreporting of febrile disease cases. Previous studies have estimated that 30–70 nonneuroinvasive disease cases occur for every reported case of WNV neuroinvasive disease (*9*). On the basis of the number of neuroinvasive disease cases reported in 2021, it is likely that 60,240–140,560 nonneuroinvasive disease cases of WNV occurred; however, only 903 (1%–2%) were reported.

### Implications for Public Health Practice

Understanding the epidemiology, seasonality, and geographic distribution of these viruses aids in clinical recognition and differentiation from other neurologic infections and guides vector control and community messaging efforts. Because there are no specific treatments for arboviral diseases, and human vaccines against domestic arboviruses are not available, prevention depends on community and household efforts to reduce vector populations,*** personal protective measures to decrease exposure to mosquitoes^†††^ and ticks,^§§§^ and blood donor screening.^¶¶¶^ Health care providers should consider arboviral infections in the differential diagnosis of aseptic meningitis and encephalitis, obtain appropriate specimens for laboratory testing, and promptly report cases to public health authorities, particularly during the summer months when most infections occur (*1*,*3*).

## Footnotes

*
https://ndc.services.cdc.gov/case-definitions/arboviral-diseases-neuroinvasive-and-non-neuroinvasive-2015

† A confirmed case meets clinical criteria for arboviral disease and has one or more of the following laboratory criteria: 1) isolation of virus from, or demonstration of specific viral antigen or nucleic acid in, tissue, blood, cerebrospinal fluid (CSF), or other body fluid, 2) a more than fourfold change in virus-specific quantitative antibody titers in paired sera, 3) virus-specific immunoglobulin (Ig)M antibodies in serum with confirmatory virus-specific neutralizing antibodies in the same or a later specimen, or 4) virus-specific IgM antibodies in CSF and a negative result for other IgM antibodies in CSF for arboviruses endemic in the region where exposure occurred.

§ A probable case meets clinical criteria for arboviral infection and virus-specific IgM antibodies in CSF or serum but with no other testing.

¶
https://www.census.gov/programs-surveys/popest.html

** 45 C.F.R. part 46, 21 C.F.R. part 56; 42 U.S.C. Sect. 241(d); 5 U.S.C. Sect. 552a; 44 U.S.C. Sect. 3501 et seq.

†† Calculated based on publicly available historic ArboNet data (1999–2022). https://www.cdc.gov/westnile/statsmaps/historic-data.html

§§
https://www2.census.gov/geo/pdfs/maps-data/maps/reference/us_regdiv.pdf

¶¶
https://www.cdc.gov/westnile/statsmaps/cumMapsData.html

***
https://www.cdc.gov/mosquitoes/mosquito-control/index.html

†††
https://www.cdc.gov/mosquitoes/mosquito-bites/prevent-mosquito-bites.html

§§§
https://www.cdc.gov/ticks/tickbornediseases/tick-bites-prevention.html

¶¶¶
https://www.cdc.gov/bloodsafety/basics.html
